# A systems biology analysis of long and short-term memories of osmotic stress adaptation in fungi

**DOI:** 10.1186/1756-0500-5-258

**Published:** 2012-05-25

**Authors:** Tao You, Piers Ingram, Mette D Jacobsen, Emily Cook, Andrew McDonagh, Thomas Thorne, Megan D Lenardon, Alessandro PS de Moura, M Carmen Romano, Marco Thiel, Michael Stumpf, Neil AR Gow, Ken Haynes, Celso Grebogi, Jaroslav Stark, Alistair JP Brown

**Affiliations:** 1 Institute for Complex Systems and Mathematical Biology, School of Natural and Computing Sciences, University of Aberdeen, Old Aberdeen, Aberdeen, AB24 3UE, UK; 2 Department of Mathematics, Imperial College London, London, SW7 2AZ, UK; 3 School of Medical Sciences, University of Aberdeen, Institute of Medical Sciences, Aberdeen, AB25 2ZD, UK; 4 Department of Biosciences, College of Life and Environmental Sciences, University of Exeter, Stocker Road, Exeter, EX4 4QD, UK; 5 Department of Microbiology, Imperial College London, The Flowers Building, London, SW7 2AZ, UK; 6 Centre for Bioinformatics, Division of Molecular Biosciences, Wolfson Building, Imperial College London, South Kensington Campus, London, SW7 2AY, UK; 7 Computational Biology, AstraZeneca, Innovative Medicines, Alderley Park, Macclesfield, Cheshire, SK10 4TG, UK; 8 Formally of the Department of Mathematics, Imperial College London, London, SW7 2AZ, UK

## Abstract

****Background**:**

*Saccharomyces cerevisiae* senses hyperosmotic conditions via the HOG signaling network that activates the stress-activated protein kinase, Hog1, and modulates metabolic fluxes and gene expression to generate appropriate adaptive responses. The integral control mechanism by which Hog1 modulates glycerol production remains uncharacterized. An additional Hog1-independent mechanism retains intracellular glycerol for adaptation. *Candida albicans* also adapts to hyperosmolarity via a HOG signaling network. However, it remains unknown whether Hog1 exerts integral or proportional control over glycerol production in *C. albicans*.

****Results**:**

We combined modeling and experimental approaches to study osmotic stress responses in *S. cerevisiae* and *C. albicans*. We propose a simple ordinary differential equation (ODE) model that highlights the integral control that Hog1 exerts over glycerol biosynthesis in these species. If integral control arises from a separation of time scales (i.e. rapid HOG activation of glycerol production capacity which decays slowly under hyperosmotic conditions), then the model predicts that glycerol production rates elevate upon adaptation to a first stress and this makes the cell adapts faster to a second hyperosmotic stress. It appears as if the cell is able to remember the stress history that is longer than the timescale of signal transduction. This is termed the long-term stress memory. Our experimental data verify this. Like *S. cerevisiae*, *C. albicans* mimimizes glycerol efflux during adaptation to hyperosmolarity. Also, transient activation of intermediate kinases in the HOG pathway results in a short-term memory in the signaling pathway. This determines the amplitude of Hog1 phosphorylation under a periodic sequence of stress and non-stressed intervals. Our model suggests that the long-term memory also affects the way a cell responds to periodic stress conditions. Hence, during osmohomeostasis, short-term memory is dependent upon long-term memory. This is relevant in the context of fungal responses to dynamic and changing environments.

****Conclusions**:**

Our experiments and modeling have provided an example of identifying integral control that arises from time-scale separation in different processes, which is an important functional module in various contexts.

## **Background**

Adaptation to dynamically changing environments is required for any life form to survive. Unicellular microorganisms often adapt to environmental changes by modulating their metabolism and reprogramming their gene expression patterns. Generally, microbes are constantly monitoring their environment via specific receptors, and then adapting their cellular physiology accordingly via changes in gene expression and metabolism that are driven by specific signal transduction networks.

### **Osmosensing in*Saccharomyces cerevisiae***

The yeast *Saccharomyces cerevisiae* is able to adapt to acute changes in extracellular osmolarity. The hyperosmotic signal is amplified and transmitted via the High Osmolarity Glycerol (HOG) signaling network, and this leads to the intracellular accumulation of the osmolyte, glycerol. Increasing intracellular glycerol concentrations decrease the intracellular water potential, restore water influx and consequently restore cell volume and turgor pressure.

**Figure 1:**
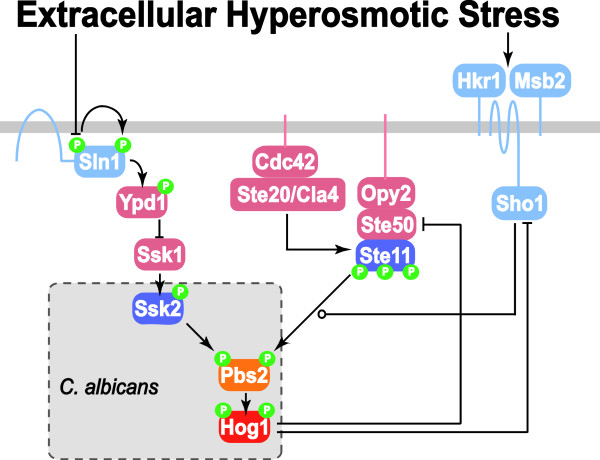
**Osmosensing networks.** The osmosensing signaling network in *S. cerevisiae* includes the Sln1 and Sho1 branches which converge at Pbs2 and eventually activate Hog1 by dual phosphorylation [11]. Under normal turgor pressure (i.e. in the absence of hyperosmotic condition), Sln1, a transmembrane protein upstream of the Sln1 branch, is autophosphorylated. This leads to phosphorylation of Ypd1, which subsequently transfers the phosphate group to Ssk1. The phosphorylated form of Ssk1 is unable to activate Ssk2 or Ssk22 via phosphorylation. Under hyperosmotic conditions, autophosphorylation of Sln1 is inhibited. This inactivates Ypd1 and consequently abrogates the inhibition of Ssk1. This is followed by the subsequent activation of the MAPKKK Ssk2 and ultimately of Hog1. Putative osmosensors Hkr1 and Msb2 that lie upstream of the Sho1 branch are postulated to directly sense the extracellular osmolarity [12]. Cdc42 interacts with and activates membrane associated Ste20 or Cla4 [13]. In addition, Cdc42 is able to bind the Ste11-Ste50-Opy2 complex (targeted to the membrane by Opy2) to bring activated Ste20 or Cla4 to their substrate Ste11 [14]. Docked with membrane-bound Sho1, activated Ste11 phosphorylates Pbs2 and eventually activates Hog1. The components of this network that are currently understood in *C. albicans* are depicted in the dotted box [15].

In *S. cerevisiae*, the HOG network has two main upstream branches, namely the Sln1 branch [1,2] and Sho1 branch [3,4] (Figure 1). These two branches converge at Pbs2, which becomes phosphorylated in response to hyperosmotic stress, eventually culminating in Hog1 activation [5]. The Sln1 branch is thought to have a linear topology (i.e. no feed-forward or feedback loops). In contrast, the Sho1 branch has at least two negative feedback control loops (Figure 1), in which activated Hog1 inhibits the activity of upstream signaling components including Sho1 [6] and Ste50 [7] via phosphorylation. In principle, such negative feedback amplifier can ensure Hog1 phosphorylation levels be kept within a narrow range upon adaptation to hyperosmotic shocks [8]. Consistent with this idea, the Sho1 branch does not respond to low hyperosmotic signals (less than 0.1 M NaCl), and displays lower maximum responses compared with the Sln1 branch [9]. In contrast, Sln1 branch operates at a high basal level in the absence of hyperosmotic signal, although the components generating such basal activation remain unidentified [9]. This basal signaling activity enables the Sln1 branch to respond faster than the Sho1 branch. Previous studies also suggest that, following osmoadaptation, Sln1 signaling is down-regulated via ubiquitin-proteosome mediated degradation of unphosphorylated Ssk1 [10].

### **Osmosensing in*Candida albicans***

In contrast to the benign model yeast *S. cerevisiaeCandida albicans* is a major fungal pathogen of humans that causes frequent mucosal infections in otherwise healthy individuals and potentially lethal infections in immunocompromised intensive care patients [16]. HOG signaling is essential for the normal virulence of *C. albicans*[17], although osmosensing in *C. albicans* is not well characterised (Figure 1). Similar to *S. cerevisiae*, in *C. albicans* osmotic stress signals are relayed to Hog1 via Pbs2 [18]. However, in *C. albicans*, osmotic stresses activate Hog1 only through Ssk2, and the Sho1 branch does not contribute significantly to osmosensing [19]. Therefore, Ssk2 appears to be the only MAPKKK that activates the HOG MAPK module under the stress conditions examined so far [19]. It is not yet clear whether the stress signal is relayed via Sln1. A novel osmotic stress signaling pathway might work in parallel with Sln1 to relay osmotic signals to Pbs2 [19]. In contrast, bioinformatic surveys of osmotic signaling components have suggested that key components of the Sho1 branch might be absent in *C. albicans* (Additional file 1: Figure S1; [20]). In summary, uncertainties remain regarding the upstream and downstream components of the HOG signaling network in *C. albicans.*

### **A systems view of HOG signaling network**

Given the importance of HOG signaling components for the virulence of *C. albicans*[17], it is important to study how this pathogen responds to osmotic shocks. In this study, we have investigated how the HOG signaling network operates in *C. albicans* using a model that encompasses the HOG signaling network and downstream adaptive processes.

Some signal transduction pathways returns to basal activity levels once a cell has adapted to environmental change. For instance, chemotaxis in *E. coli* is achieved through a sensing network whose activity always returns to the basal levels once the cell moves towards the attractant or away from the repellent [21]. Also, the phosphorylation status of the heat shock transcription factor returns to basal levels once *C. albicans* cells have adapted to elevated ambient temperatures [22]. This type of “perfect adaptation” may be significant in minimizing the potential impact of signaling crosstalk. In particular, the HOG pathway shares common components with the pheromone sensing pathway and the filamentous growth pathway. Perpetual HOG signaling activity might inappropriately activate other pathways.

**Figure 2:**
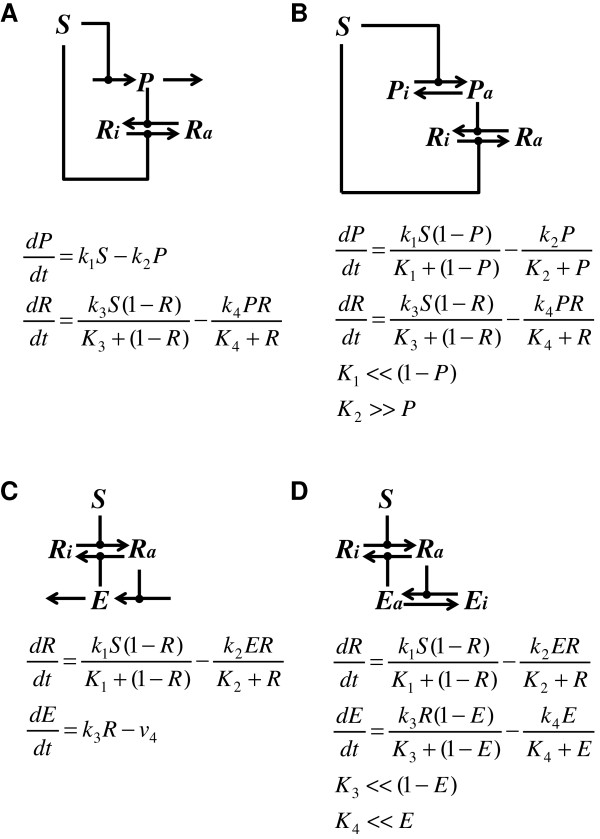
**Networks that enable response (R) to perfectly adapt to signal (S). (A)** Incoherent feedforward loop: on one hand, *S* directly phsophorylates and hence activates *R*; on the other hand, *S* inactivates *R* proportionally via stimulating the expression of proportioner (*P*) that dephosphorylates *R*. The activation and inactivation effects cancel out for *R* in such a loop. As a result, *R* always resumes to the original state upon adaptation. In the mathematical model, R refers to the phosphorylated form of *R* (i.e. *Ra* in the diagram). The first equation dictates that the steady state expression level of *P* is proportional to *S* (this is why it is called “proportioner”). Plug this relationship into the second equation and take the steady state: *S* in the first term will cancel with *P* in the second term. This makes the steady state of *R* independent from *S*. **(B)** Incoherent feedforward loop. This slightly more complex example also helps demonstrate perfect adaptation. In this case, *S* phosphorylates and thereby activates both *R* and *P*. If *S* operates in the saturated regime (i.e. *K*_*1*_ < <(*1-P*)) and the dephosphorylation of *P* operates in the unsaturated regime (i.e. *K*_*2*_> > *P*), then the first equation reduces to the same form as that in **(A)**. By the same reasoning, *Ra* in this incoherent feedforward loop perfects adapts. **(C)** Negative feedback loop involving time-scale separation: *S* phosphorylates and activates *R* that subsequently simulates the expression of its own inhibitor *E*. In the model, *R* refers to the phosphorylated form of *R* (i.e. *Ra* in the diagram). When *E* disappears at a constant rate *v*_*4*_, the second equation ensures that the steady state *R* is independent of *S*, and is only determined by *k*_*3*_ and *v*_*4*_. In this case, *E* integrates over *R*, and this negative feedback loop constitutes an integral controller. As a special case, when the half life of *E* is much longer than the time scale of other reactions, *v*_*4*_ is approximated to 0. Consequently, *R* always resumes 0 upon adaptation. **(D)** Negative feedback loop involving saturated enzyme kinetics: in this slightly more complex scenario, element (*E*) is the phosphatase of *R* and is activated by *R* via phosphorylation. If *Ra* and *E*’s phosphatases both operate in their saturated regimes, then the second equation reduces to the same form as that in **(C)**. By the same reasoning, such a negative feedback loop is capable of perfect adaptation.

**Figure 3:**
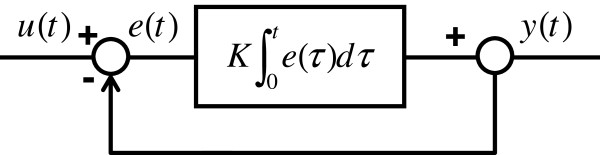
**Block diagram of integral control.** For a system, the input signal is *u*(*t*), and the output signal is *y*(*t*). The difference between *u*(*t*) and *y*(*t*) is defined as the error signal *e*(*t*).

A recent *in silico* study reveals that only two network topologies ensure perfect adaptation, incoherent feed-forward loop and negative feedback loop ([23]; see Figure 2 for detailed discussions). In incoherent feed-forward loop, the response element is subject to a positive regulation that is proportional to a negative regulation simultaneously. The overall outcome is that the final output is insensitive to the signal. The negative feedback loop involves integral control, a regulatory mechanism whereby the controller output is proportional to the amount and duration of the error signal (the “error signal” being defined as the difference between a signaling network’s current state and its final adapted state) (Figure 3). Integral control enables the cell to convert an error signal into the temporal integral of the activity of the signaling pathway. In other words, the output of the signaling network (i.e. changes in the downstream adaptive processes) will be proportional to the temporal integral of the signaling pathway. Since the information is encoded as the temporal integral of the signaling pathway, the signal transduction pathway is able to “integrate out” the error signal, returning to basal levels once the cell has adapted to its new state. Hence, integral control provides a means of achieving the perfect adaptation of a signal transduction pathway.

The integral controller employs the time integral of the error signal *K* ∫ _0_^*t*^*e*(*τ*)*dτ* as the feedback mechanism.

Interestingly, integral control mechanisms have arisen frequently in evolution, presumably because they provide robust means by which systems can adapt effectively to constant environmental stimuli despite stochasticity. Indeed, systems biology studies have revealed a variety of biological processes that employ integral control mechanisms including bacterial chemotaxis [24], calcium homeostasis [25] and energy metabolism [26].

Elegant single cell analyses of nuclear Hog1 enrichment in *S. cerevisiae* following hyperosmotic shock have revealed that nuclear Hog1 levels return to basal levels once the cell has adapted to hyperosmotic conditions, and therefore that the HOG system displays perfect adaptation [27]. These authors showed that upon exposure to osmotic stress, the time integral of Hog1 activity is linearly related to the increase in extracellular osmotic pressure within a certain range [27]. This verifies that Hog1 exercises integral control over glycerol production in response to a persistent hyperosmolarity. This key discovery offers a starting point for developing simple mathematical models of osmotic stress responses in *S. cerevisiae* and *C. albicans*.

Using modeling and experimentation, we demonstrated that such integral control arise through a separation of the time scales between rapid signaling events and the relatively slower downstream adaptive processes. In addition, a hyperosmotic shock triggers long-term changes in the physiology of yeast cells that prepares them for subsequent hyperosmotic shocks. It appears as if a yeast cell can “remember” a previous hyperosmotic shock over a period that is longer than the time scale of signal transduction, and therefore adapts quickly to subsequent hyperosmotic shocks. We term this phenomenon a long-term stress memory. Our model suggests the HOG system also has a short-term memory that is engendered in the activation and inactivation of the intermediate kinases during the signal relay. The short-term memory might be affected by the regulation of glycerol channels. Our work highlights the need to study the properties of a signal transduction network in an appropriate biological context.

## **Results**

### **Cellular memory and model formulation**

**Figure 4:**
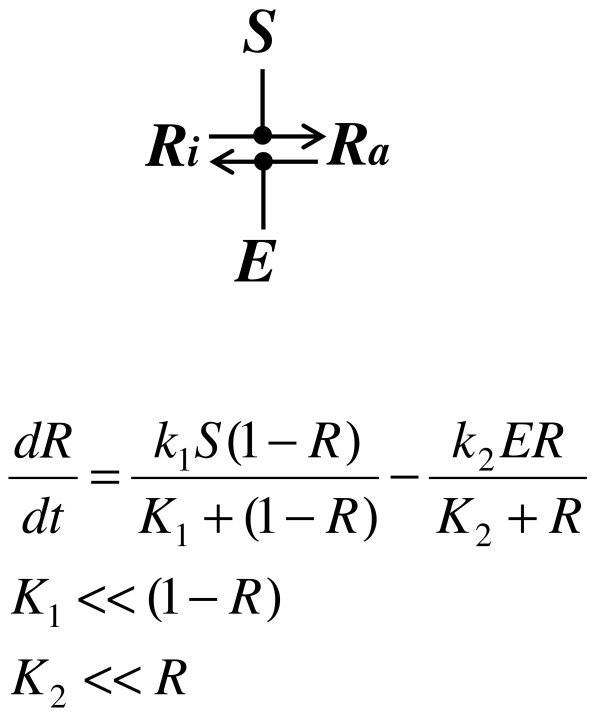
**Integral control could result from saturation enzyme kinetics.** Here, both kinase and phosphatase operate in the saturated region, the equation governing *R* reduces to dRdt=k1S−k2E. Therefore, *R* integrates the difference between *S* and *E*.

Several possible mechanisms enable integral control (Figure 2C-D, Figure 4). Our aim was to test which of these mechanisms operates during osmoadaptation. Consider a signaling molecule that exists in either a phosphorylated or unphosphorylated state (Additional file 1: Figure S2). Its kinase activity is modulated by a signal, while its phosphatase has a constant activity. Given a constant signal, if the kinase and phosphatase both operate in the saturation regimes (i.e. the concentrations of the kinase and the phosphatase far exceed their respective K_m_ values), using a total quasi-steady-state approximation to describe the phosphorylation and dephosphorylation processes, it can be demonstrated that the activity of this signaling molecule is mainly determined by the activities of its kinase and phosphatase ([27,28], for details see Figure 4). Thus, the activity of this signaling molecule effectively integrates the difference between its kinase and phosphatase activities. In addition, this mechanism involving saturated enzyme kinetics would result in a memory whose duration is determined by the rates at which the kinase and the phosphatase activities change. This is predicted to endow a short-term memory that lasts seconds because changes in the kinase and phosphatase activities typically are rapid [28]. However, it is unlikely that any component in the HOG pathway would operate under both saturating phosphorylation and dephosphorylation conditions because of the low concentrations of MAPK pathway components in *S. cerevisiae* (see supplementary material section 2.1 for details). An alternative mechanism of saturated enzyme kinetics can give rise to perfect adaptation is discussed in Figure 2D. For the same reason as the previous case, it would leave only a short-term memory.

An alternative mechanism by which integral control can arise is via differential time scales in biological processes (see Figure 2C for details). Phosphorylated Hog1 activates several parallel mechanisms to increase the intracellular levels of glycerol in *S. cerevisiae*. Active Hog1 up-regulates the expression of genes encoding glycerol biosynthetic enzymes (*GPD1GPD2* and *GPP2*). Hog1 also increases the activity of a key enzyme (Pfk26) that diverts metabolic flux towards glycerol production [29]. Furthermore, Hog1 stimulates the production of Stl1, a component of the glycerol/H^+^ symport system that assimilates glycerol from the growth medium [11,30]. In principle, if the timescale for the inactivation any of these mechanisms is much longer than the timescale for achieving adaptation to higher osmolarity, then we can safely ignore the inactivation of the adaptive processes during the adaptation process. Since the glycerol channel activities change rapidly in response to external osmotic pressure [27], the adaptive processes we consider here are only related to glycerol production. Hence, if inactivation of glycerol production is a much slower process than adaptation itself, then the change in glycerol production rate (i.e. the output of the HOG signaling network) is determined by the time integral of phosphorylated Hog1. Indeed, in *S. cerevisiae* the rate of increase in glycerol biosynthetic enzyme levels is proportional to the time integral of Hog1 phosphorylation [31]. This change in physiological status would be likely to generate a molecular memory whose duration is determined by the rate at which the glycerol production rate reduces to basal levels following a return to hypo-osmotic conditions. Therefore, a significant memory was predicted because the degradation of stable enzymes is relatively slow. A descriptive model detailing Hog1 integral control was experimentally validated [31]. However, the idea that this integral control arises in *S. cerevisiae* through a separation of time scales between signaling and glycerol production rate changes was not fully recognized. The connection between Hog1 adaptation and integral control was not reported till later [27].

Whilst upstream components of HOG signaling have diverged significantly in the evolutionarily divergent yeast, *C. albicans*[19,20], Hog1 integral control of osmotic stress adaptation might be conserved. Therefore our first aim was to build a simple mathematical model that highlights Hog1 integral control for both *S. cerevisiae* and *C. albicans*, and then use it to test alternative mechanisms by which a long-term memory can arise.

### **Model construction**

**Figure 5:**
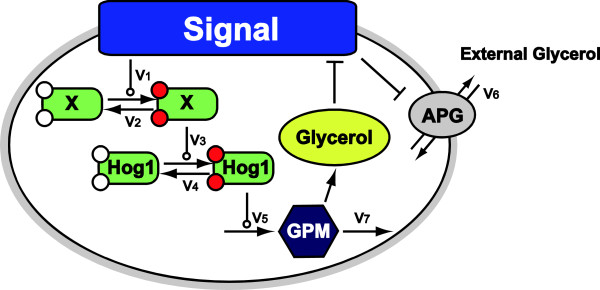
**Overview of the model.** The hyperosmotic stress signal is represented as the change in the difference between intracellular and extracellular osmotic pressures (i.e.*S*(*t*) = (*Π*_*i*_^0^ − *Π*_*e*_^0^) − [*Π*_*i*_(*t*) − *Π*_*e*_(*t*)]; *Π*_*i*_^0^: initial intracellular osmolarity, *Π*_*e*_^0^: initial extracellular osmolarity, *Π*_*i*_(*t*): current intracellular osmolarity, *Π*_*e*_(*t*) current extracellular osmolarity). Thus, the initial signal is equivalent to the increase in osmolarity caused by the extracellular NaCl. Upon an increase in extracellular osmolarity, the signal becomes positive and activates an intermediate kinase (v_1_) and then Hog1 (v_3_), both of which are balanced by dephosphorylation activities (v_2_, and v_4_ respectively, associated kinetic parameters are constant). Once activated, Hog1 induces the activity of glycerol production machinery (GPM) (v_5_) which produces glycerol. Intracellular glycerol passively diffuses out of the cell through an aquaglyceroporin (AGP), driven by the concentration gradient across cell membrane (v_6_). Hyperosmotic shock triggers rapid closure of the aquaglyceroporin to retain glycerol, while a hypoosmotic condition causes the aquaglyceroporin to open to a higher degree than that of the steady-state (v_6_) and triggers a rapid decrease in the glycerol biosynthesis rate (v_7_). In both *C. albicans* and *S. cerevisiae*, increased intracellular glycerol concentration elevates the intracellular osmotic pressure and eventually attenuates the signal, indicating adaptation to the new condition.

In our model, a pathway or module describing a certain biological function is represented by a single reaction, encapsulating the main dynamical features of several molecular events. Our model encompasses the HOG signaling network that transmits osmotic stress signal and modulates Hog1 activity (v_1_ to v_4_), Hog1-dependent intracellular glycerol accumulation (v_5_ and v_7_) and an osmolarity-regulated AquaGlyceroPorin activity (v_6_) (AGP, i.e. Fps1 in *S. cerevisiae*; unidentified in *C. albicans*) (Figure 5). An acute environmental hyperosmotic shift is proposed to cause rapid closure of this aquaglyceroporin to reduce glycerol efflux [27]. It is generally believed that this is a fast change mediated by a rapid conformational change of the aquaglyceroporin under hyperosmotic conditions, and that the aquaglyceroporin will reopen once the cell has adapted [11,32].

In our model we also assume that glycerol productivity does not decrease under hyperosmotic conditions (v_7_ = 0). Thus, the increase in the activity of Glycerol Production Machinery (GPM) will be proportional to the time integral of phosphorylation levels of Hog1. We also note that in the long term in *S. cerevisiae*, active Hog1 is reported to phosphorylate the aquaglyceroporin to target it for subsequent degradation [11,32]. However, this regulation has yet to be quantified in *S. cerevisiae*, and it is not known whether the aquaglyceroporin in *C. albicans* is regulated in the same way. Hence, the degradation of aquaglyceroporin, despite its potential interest, is not included in our model.

**Figure 6:**
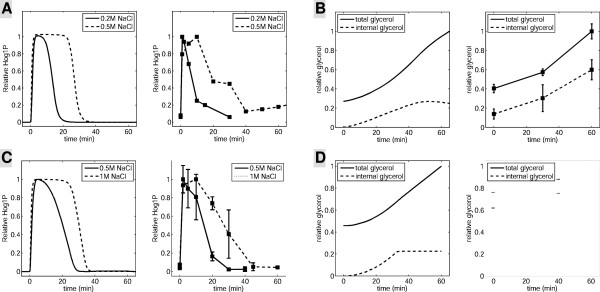
**Experimental measurements and model construction.** Wild-type *S. cerevisiae***(A, B)** and *C. albicans***(C-D)** cells were exposed to hyperosmotic condtions. **(A)***S. cerevisiae*: 0.2 and 0.5 M NaCl; **(B)***S. cerevisiae*: 1 M NaCl; **(C)***C. albicans*: 0.5 and 1 M NaCl. **(D)***C. albicans*: 1 M NaCl. Hog1 activity, intracellular glycerol and total glycerol were measured in time courses of up to 1 hour. Experimental data for *S. cerevisiae* in A and B are published results [31]. *C. albicans* data are from this study. Model predictions were plotted separately from experimental measurements.

To construct our model for both *S. cerevisiae* and *C. albicans*, we investigated the dynamics of different physiological variables for various doses of NaCl. These included Hog1 activity, and intracellular and extracellular glycerol concentrations. *S. cerevisiae* data were taken from several publications [31,33,34], and we systematically measured these values in *C. albicans* (Figure 6). As discussed in supplemental section 2 in details, most kinetic parameters were set to reproduce the dose–response in Hog1 activation. The rest were manually tuned with respect to the single shock experiment.

### **The simple model explains published data**

[27] investigated the dynamics of Hog1 activation by monitoring the nuclear enrichment of fluorescently-tagged Hog1 (Hog1-YFP) in individual *S. cerevisiae* cells following hyperosmotic shock. They defined the nuclear enrichment of Hog1 as the relative change in the proportion of nuclear localised Hog1-YFP compared with pre-stimulus levels [27]:

(1) Hog1enrichement=FnucFcelltFnucFcell0−1

where *F*_*nuc*_ is nuclear fluorescence signal, *F*_*cell*_ is whole cell fluorescence.

They found that the time integral of nuclear Hog1 enrichment increases linearly with extracellular osmolarity [27]. It was subsequently shown in a computational study that the nuclear enrichment of Hog1 is highly correlated with the Hog1 phosphorylation level [35]. This implies that the time integral of Hog1 phosphorylation is linear with extracellular osmolarity:

(2) $$k\int_{0}^{t}Hog1P(t)dt=\Pi_{\mathrm{NaCl}}$$

Our model is consistent with this. First, following adaptation to the higher osmotic pressure, the rate of production of intracellular glycerol is then balanced by glycerol efflux so as to maintain constant intracellular glycerol levels. In principle, three factors contribute to glycerol efflux rate: [i] intracellular glycerol levels, [ii] extracellular glycerol levels, and [iii] aquaglyceroporin activity.

(3) $$v_{6}=c_{G}\cdot \left(G_{i}-\frac{G_{e}}{V_{e}}\right)$$

We argue that upon adaptation, intracellular glycerol concentration is the main factor that determines glycerol efflux. Firstly, in our experimental conditions for *C. albicans* and those reported in the literature for *S. cerevisiae*, intracellular glycerol concentration *G*_*i*_ is at least 3-order of magnitudes higher than its extracellular concentration GeVe, mainly because extracellular space *V*_*e*_ is much larger than cellular volume [33]. Thus, the concentration difference that drives the passive efflux of glycerol is mainly determined by intracellular glycerol concentration, and is not significantly affected by extracellular glycerol concentration. Secondly, we assume that the activity of aquaglyceroporin *c*_*G*_ is regulated by the hyperosmotic signal:

(4) $$c_{G}=max(-k_{\mathrm{AGP}}\cdot S+b_{\mathrm{AGP}},0)$$

This signal reduces to a small value following cellular adaptation. This suggests that the activity of aquaglyceroporin will return to its resting value *b*_*AGP*_ after adaptation. Taken together, the rate of glycerol efflux is determined by and is proportional to the intracellular glycerol concentration *G*_*i*_ alone. Because glycerol production *P*_*G*_ balances glycerol efflux *v*_***6***_, the former is also proportional to intracellular glycerol concentration *G*_*i*_.

The increase in intracellular osmolarity caused by the rise in the intracellular glycerol concentration *G*_*i*_ eventually matches the new extracellular osmolarity ∏ _*NaCl*_ to reduce the signal upon adaptation. Thus, increase in the extracellular osmolarity that triggered the response ∏ _*NaCl*_ is linearly proportional to the increase in glycerol production rate *P*_*G*_, which is determined by ∫ _0_^*t*^*Hog*1*P*(*t*)*dt* due to the integral control. Hence, the time integral of the nuclear enrichment of Hog1, which is highly correlated with the phosphorylation of Hog1, has a linear relationship with ∏ _*NaCl*_.

### **Hyperosmotic responses in* C. albicans***

In response to hyperosmotic conditions, *S. cerevisiae* closes the aquaglyceroporin channel, Fps1, to prevent glycerol leakage and help to maintain a high intracellular glycerol concentration [27,31]. Similarly, *C. albicans* appears to have a high restriction over glycerol efflux by an aquaglyceroporin. Our experimental data show that, in the presence of 1 M NaCl, the increase in total glycerol (i.e. the sum of intra- and extra-cellular glycerol) is mainly due to the induction of intracellular glycerol, and extracellular glycerol (i.e. the difference between total and intracellular glycerol) does not change significantly (Figure 6D). This is consistent with the idea that the aquaglyceroporin closes quickly in response to hyperosmotic stress thereby inhibiting glycerol leakage. This would maximize the efficiency with which cells could accumulate glycerol. However, we note that the experimental data suggest that intracellular glycerol does not plateau upon Hog1 adaptation (Figure 6B6D).

Our measurements of intracellular and total glycerol concentrations revealed relatively high initial intracellular glycerol concentrations in unstressed cells, which were induced about four-fold following a hyperosmotic shock. Our experimental procedures, which examined exponential cells grown in rich media at 30°C, were designed to minimize environmental stress. We would point out that the glycerol assay does not have the sensitivity to measure low concentrations of glycerol accurately [33]. Nevertheless, our data indicate that under our experimental conditions unstressed *C. albicans* cells contain intracellular glycerol (Figure 6D). In our model we assume that the intracellular glycerol concentration is in equilibrium with the extracellular glycerol concentration pre-stress.

Our experimental data revealed two significant differences between the responses of *C. albicans* and *S. cerevisiae*. Firstly, in *S. cerevisiae* the proportion of phosphorylated Hog1 reaches its maximum value when concentrations of NaCl are greater than or equal to about 0.2 M (Figure 6A), whereas in *C. albicans* the percentage of phosphorylated Hog1 continues to increase, even in the presence of 0.5 M NaCl (data not shown). The NaCl concentration at which Hog1 phosphorylation amplitude becomes saturated was used to constrain parameters of the HOG signaling pathway (for details see supplemental information, section 2.1). Secondly, in *S. cerevisiaeGPD1* expression was strongly induced at the protein level and this regulation was mediated by Hog1 [31]. However, in *C. albicansGPD1* is expressed at high levels even in the absence of hyperosmotic stress, and Gpd1 is not highly induced under hyperosmotic conditions [36]. However, expression of *GPD2* was found to be induced at the mRNA level [36]. This indicates differential regulation of the Gpd isoenzymes by Hog1 in *C. albicans*.

### **Model validation**

As mentioned previously, the existence of a long term memory is the key to distinguishing the mode of control that Hog1 exerts upon glycerol production (i.e. proportional, derivative or integral) and to explain its underpinning mechanism (i.e. saturated enzymatic kinetics or the separation of time scales). The existence of a long-term cellular memory would imply a mechanism of Hog1 integral control that arises through the separation of time scales between Hog1 signaling events and glycerol production rate changes.

**Figure 7:**
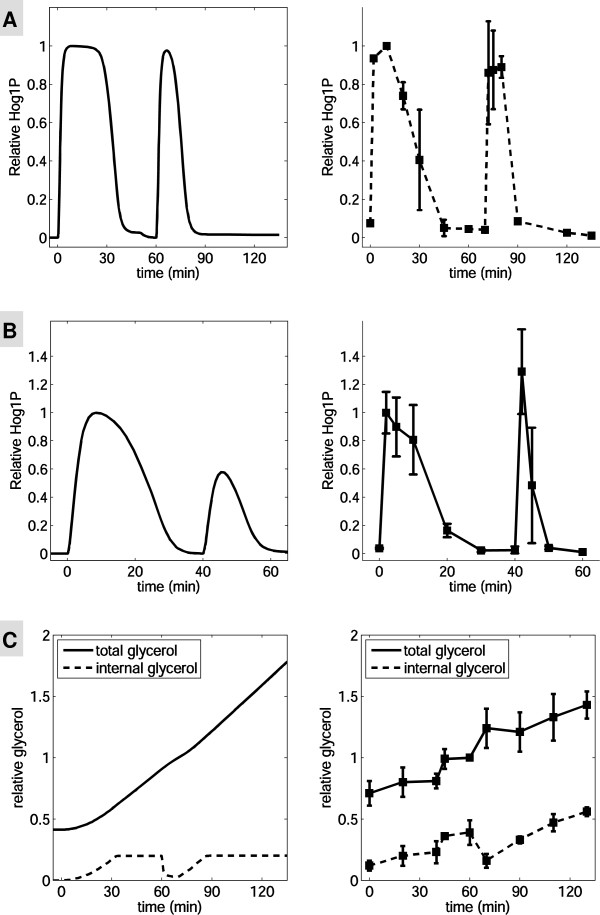
**Responses of*C. albicans*to repeated hyperosmotic stress.***C. albicans* cells were exposed to two identical hyperosmotic shocks (A: 1 M NaCl; B: 0.5 M NaCl). After adaptation to the first shock (A: 60 min; B: 30 min), the cells were shifted to medium lacking NaCl for 10 min. Subsequently, the cells were subjected to an identical second shock (A: 1 M NaCl, 60 min; B: 0.5 M NaCl, 30 min). Hog1 phosphorylation was measured at various time points over a 1 or 2 h time course in duplicate **(A)** or triplicate **(B)**. Experimental and simulated Hog1-phosphorylation levels are normalized against the first Hog1 phosphorylation peak. **(C)** Relative total and intracellular glycerol concentrations (0.5 M NaCl) were measured at various time points over a 2 h time course (2 to 6 replicates were measured up to 60 min, and in duplicate after 60 min). Both experimental and simulation results are normalized to the total glycerol concentration at 60 min.

To test this, we developed an experimental assay whereby *C. albicans* cells were exposed to an initial hyperosmotic shock (1 M NaCl) until they fully adapted. These cells were then removed from the hyperosmotic condition for about 10 min, and were then exposed to an identical, second hyperosmotic shock. We found that the duration of Hog1 phosphorylation in the second response was much shorter than that for the first stress (Figure 7A). This provided direct experimental evidence that adaptation to 1 M NaCl results in a memory that lasts for at least 10 min. Our model, manually turned to reproduce single-shock data, reproduces these results well quantitatively. We also assayed the time course of Hog1 phosphorylation during sequential hyperosmotic shocks of 0.5 M NaCl. Once again the model predictions were in good agreement with experimental results (Figure 7B).

We then tested whether this molecular memory was due to the retention of a relatively high glycerol synthetic capacity after adaptation to the first hyperosmotic stress. This was achieved by monitoring the intracellular glycerol levels generated by *C. albicans* cells after repeated exposure to hyperosmotic conditions (Figure 7C). The data showed that intracellular glycerol levels plateau within the first 45 min, which was coincident with the disappearance of Hog1 phosphorylation during the first hyperosmotic shock. Intracellular glycerol levels were then restored within 20 min of the second shock, also coincident with the disappearance of Hog1 phosphorylation. This provided further evidence that *C. albicans* cells retain high glycerol productivity for some time after the first adaptation. It also strongly supports the idea that the integral control in the HOG system is endowed by the separation of time scales. We note that the total glycerol was induced about 2-fold while it is simulated to increase 4-fold. However this minor discrepancy does not change the main conclusions drawn above.

### **Duration of long-term memory**

**Figure 8:**
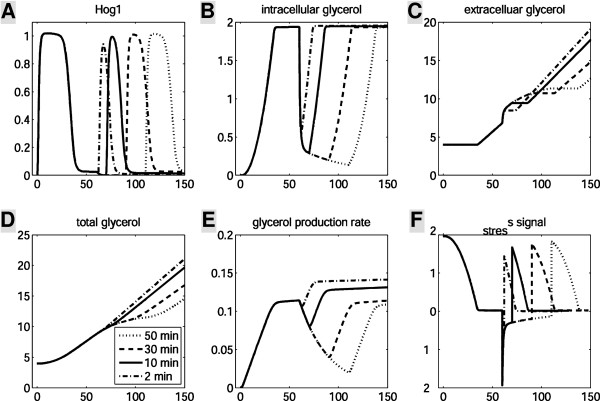
**Simulations of the duration of cellular memory following exposure of*C. albicans*to 1 M NaCl.** Cells are stressed for 60 min, undergo an interval without stress before a second identical stress is imposed. The interval varies between 2 to 30 min, as indicated in the lower left inset. **(A)** Hog1 is plotted in relative values. **(B)** Internal glycerol concentration is in M. **(C-D)** Extracellular and total glycerol concentration are in mM. **(E)** Glycerol production rate is in M/min. **(F)** Stress signal has a dimension of Osm.

Having shown that *C. albicans* displays an osmotic stress response memory and discussed its relevance to integral control, we used our model to make predictions about the behaviour of this memory. Our model predicted that during the hypoosmotic period between the first and second hyperosmotic shocks, cells would rapidly release the intracellular glycerol that was accumulated during the first hyperosmotic shock. We also predicted that the proteolytic degradation of the glycerol production enzymes synthesized during first hyperosmotic shock might take a relatively long time. These factors influence how long the memory lasts. By changing the duration of the interval between two consecutive hyperosmotic shocks, our model predicted that a short interval, such as 2 min, would not result in significant reduction in the glycerol production rate (Figure 8E). We therefore inferred that the rapid closure of the aquaglyceroporin would be sufficient to maintain the intracellular glycerol level when the second hyperosmotic shock is applied. Interestingly, the model also predicted that even though the intracellular glycerol concentration is fully restored during the second adaption, the glycerol production rate might not (dashed line in Figure 8E). During a longer interval between hyperosmotic shocks the glycerol production machinery is predicted to decrease significantly. In this case the second hyperosmotic adaptation would require a higher glycerol production rate and hence more sustained Hog1 activation. For example, after a gap of 30 min between shocks, the *C. albicans* cell is predicted to return to its pre-shock physiology and respond to the second shock in nearly the same way as it did to the first stress (dotted line in Figure 8A, E).

### **Long-term memory impacts on short-term memory**

The HOG signaling networks in *S. cerevisiae* and *C. albicans* also possesses a “short-term memory” of hyperosmolarity. This results from the time it takes for the signaling components to be inactivated via dephosphorylation/phosphorylation. In response to a fluctuating osmotic signal, if the duration of the “off” phase of the signal is longer than the time required to inactivate the signaling components on HOG pathway, the phosphorylation levels of Hog1 would be predicted to follow the fluctuating signal. However, if the duration of the “off” phase is shorter than the inactivation time of the HOG pathway, the HOG network would remain active into the next cycle. The short-term memory of HOG pathway (i.e. the inactivation time for HOG pathway) typically lasts several minutes [37,38].

To better understand this phenomenon, we developed a theoretical framework for the basic HOG signaling module, which is represented by a protein that is phosphorylated and dephosphorylated by its kinase and phosphatase, respectively. Employing Michaelis-Menten kinetics, we show that this module is essentially a low-pass filter (supplemental section 2.2). In other words, given a constant phosphatase level, the phosphorylation level of the protein faithfully follows a low-frequency activation signal while integrating a high-frequency signal, as reported previously [28,37,38]. We also derived analytically how the frequency of an activation signal would impact upon the phosphorylation level of an enzyme (see supplemental section 2.2 for details of the solution). In this way, our analytic solution of the frequency response has furthered our understanding of the behaviour of this simple module. In addition, we showed that in response to an alternating signal, the amplitude of the output of a network decreases with the number of cascades it contains (supplemental section 2.2). This ability to attenuate high frequency signals would allow cells to filter out random fluctuations (white noise) in environmental osmolarities, whilst maintaining its ability to sense significant environmental changes over longer time scales.

**Figure 9:**
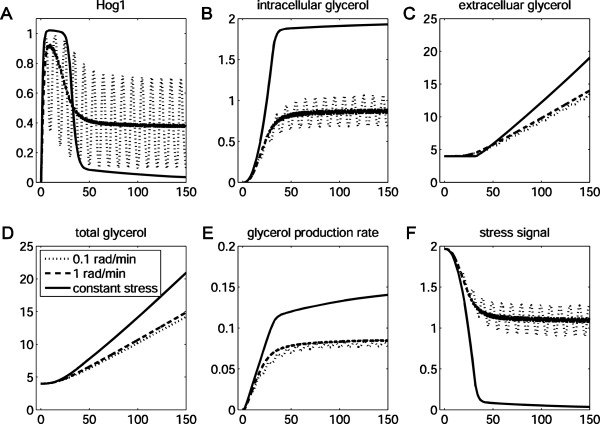
**Predictions of frequency responses in*C. albicans*.** Responses of a *C. albicans* cell to a sinusoidal osmotic signal with peak-to-peak amplitude of 1 M NaCl (centered at 0.5 M NaCl) at different frequencies are simulated (dotted line: 0.1 rad/min; dashed line: 1 rad/min; real line: constant stress).

These studies were carried out using theoretical frameworks that only modeled the signal transduction pathway itself. We then extended our analyses to include downstream adaptive processes by simulating how the entire system responds to a fluctuating signal. As previously mentioned, the HOG signaling network faithfully monitors low frequency signals. As expected, our simulation results suggest that Hog1 phosphorylation levels follow the sinusoidal signal when its frequency is 0.1 rad/min (Figure 9A). However, the average of Hog1 phosphorylation initially decreases to a constant value. This is due to the sequential accumulation of intracellular glycerol during each period. Cells that have acquired relatively high intracellular glycerol concentrations are essentially pre-adapted to a hyperosmotic shock posed by 1 M NaCl.

Meanwhile, the HOG signaling network integrates high frequency signals. It is expected that a cell will respond to a high frequency sinusoidal signal in a similar manner as to a constant hyperosmotic stress with the time-average amplitude of the sine signal. In addition, due to the integral control, Hog1 phosphorylation levels might be expected to return to basal levels once the cell has adapted. However, our simulations predict that Hog1 phosphorylation does not return to the initial condition. Instead the model predicts that, due to the quick release of glycerol in the “off” phase of the signal, the cell does not accumulate intracellular glycerol to the same level as it does under a constant signal (Figure 9B), and the glycerol production rate does not reach the same level as that under a constant stress (Figure 9E). This would be similar to the molecular response of mutants that are unable to close the aquaglyceroporin channel, which must constantly activate Hog1 to maintain a high intracellular glycerol concentration. Based on this consideration, we designed an asymmetric square wave signal with the same frequency of 1 rad/min, but where each “on” phase is longer than each “off” phase. The simulations predicted that under these conditions, Hog1 phosphorylation levels will recover when the “off” phase is sufficiently short (see supplement section 2.6 for details). These results suggest that the frequency of the cellular response is dependent upon both the short-term memory in the signaling pathway (i.e. the low-pass filter) and the property of glycerol channels. The results highlight the need to place a model of the signal transduction network into an appropriate context when studying its properties.

**Figure 10:**
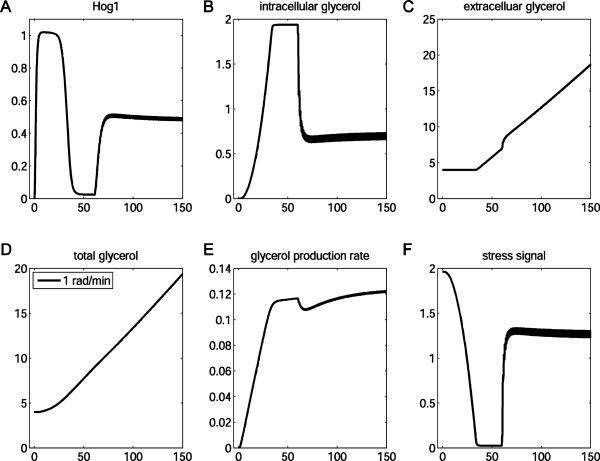
**Long-and short-term memories in*C. albicans*.** A cell initially undergoes a constant hyperosmotic shock of 1 M NaCl within the first 60 min. This is followed by the treatment of a 1 rad/min sinusoidal signal with a peak-to-peak amplitude of 1 M NaCl centered at 0.5 M NaCl.

We also investigated whether the long-term memory, a property of the adaptive process itself, affects the short-term memory of the signaling pathway. We performed simulations of a cell pre-stressed in 1 M NaCl for 60 min and observed how it then reacted to a 1 rad/min sinusoidal osmotic signal of a peak-to-peak amplitude of 1 M NaCl centred at 0.5 M NaCl (Figure 10). The model predicted that it takes much shorter for the Hog1 activity to adapt to similar levels compared with those cells excited by the same signal but without a preconditioning history (compare Figure 10 with the dashed lines in Figure 9). This indicates that, although HOG signaling behaviour is typically conceived to be determined by the short-term memory alone, long-term memory also contributes to signaling behaviour. Our computational study suggests that a pre-stressed cell possesses relatively high levels of intracellular glycerol that offset the environmental osmotic shock. Hence, as a result of this long-term memory, a stronger osmotic shock is required to achieve an equivalent signaling amplitude to non-pre-stressed cells. Also, this effect partially overrides the short-term memory, at least in over short time scales. This insight again emphasises the importance of a systems approach to study the properties of signal transduction pathways in vivo.

## **Discussion**

Recently, Gennemark and coworkers reported a simple mathematical model of hyperosmotic responses in *S. cerevisiae*[33]. Their model concisely described the biophysical changes of a hyperosmotically stressed yeast cell, and related those changes to glycerol production. This model is able to reproduce some published data on the changes in intracellular and extracellular glycerol concentrations in response to different physiological perturbations. Instead of integral control, their model assumes a time-delayed proportional control of glycerol production, i.e. glycerol productivity at a particular time is assumed to be proportional to the change in turgor pressure (relative to a constant reference turgor pressure). A consequence of this assumption is that there should be no memory effects in the stress response dynamics. This motivated us to investigate whether a model including integral control would be more appropriate.

Also inspired by the recent finding of Hog1 integral control in *S. cerevisiae*[27], we investigated osmotic stress responses in *C. albicans* by constructing a low granularity mathematical model assuming Hog1 integral control. This model integrates the HOG signal transduction pathway with downstream adaptive processes, and encompasses both hyper- and hypo-osmotic conditions. In this model, a signaling or biochemical pathway was concisely represented by an overall reaction (Figure 5). Hence, the property of the pathway was defined by the associated kinetic parameters. The model was calibrated using in-house generated time-course data of Hog1 phosphorylation plus intracellular and total glycerol levels following exposure to two doses of NaCl. Subsequently, Hog1 integral control was experimentally verified in *C. albicans* using experiments in which cells were subjected to repeated NaCl insults. The data showed that pre-stressed cells adapted to a second hyperosmotic shock more quickly, as if they remembered the hyperosmotic history. This phenomenon is known as the “long-term stress memory”, because its duration was longer than the time required for signal transduction to take place. Using this model, we reasoned that the cellular memory observed under hyperosmotic conditions is a hallmark of the separation of time scales between HOG signaling and reduction of glycerol production rates. In this case this mechanism enables Hog1 integral control.

The separation of time scales in different biological processes is a universal phenomenon. Often, signaling networks that perceive environmental conditions such as nutrient availability, chemical insults, and mating pheromones are required to function at much faster rates (i.e. seconds) than the ensuing regulatory networks which encompass transcriptional and translational controls (i.e. tens of minutes). It is also common for signals to be converted, via modulation of transcriptional regulators, into the expression of a stable product (i.e. a stable mRNA or protein). Such a separation of time scales could result in an integral control mechanism that would generate an experimentally testable long-term memory. Therefore our experimental approach could be further applied to other signaling networks to examine such a property. In addition, the identification of such a phenomenon allows mathematical simplifications of the regulatory network under study, an important step towards a thorough understanding of signaling networks which may be obscured in exhaustive descriptive models.

The teleological question as to why the yeast cell employs an integral control mechanism remains to be answered. Among the various types of feedback mechanism, proportional control and derivative control mechanisms would allow a system to adapt to a new status that is different from the original condition. However, integral control ensures perfect adaptation of the system, whereby signaling returns to basal levels once adaptation is achieved. This would reduce the potential for cross-talk between signaling pathways. The ability of the cell to restore turgor pressure in the face of an osmotic challenge is crucial for the restoration of growth. Should Hog1 signaling regulate glycerol production via proportional control, a change in osmotic pressure would be reflected by a proportional change in Hog1 activity. In other words, the ability to sense diverse amplitudes of osmotic signal would require high concentrations of HOG signaling proteins, the production of which would demand considerable cellular resources. In contrast, the integral control mechanism converts the error signal into the temporal integral of HOG activity. This avoids the need to express HOG pathway components at high levels. In fact in *S. cerevisiae*, Hog1 phosphorylation levels become saturated in response to relatively low hyperosmotic signals. In addition, derivative control offers gradual changes in a system, which might be too slow to generate the necessary adaptation to external osmotic challenges. Indeed, derivative control is often found to provide a buffering capacity in cellular systems. For instance, in energy metabolism, phosphorylated molecules, such as phosphocreatine, act as a buffer during high energy demand periods. Phosphocreatine implements a biological derivative control over ATP concentration [26].

Our modeling further suggests that the frequency response of the HOG signaling pathway can be affected by the regulation over the glycerol channels. This prediction is significant because this frequency response is generally thought to be a property of the short-term memory mediated by the regulation of intermediate protein kinases alone. In addition, our simulations show that the long-term memory may also affect the way a cell responds to a high frequency signal. Our simulations suggest that a cell that has previously adapted to hyperosmolarity retains relatively high intracellular glycerol levels for a period. Consequently, the high intracellular osmolarity counterbalances the hyperosmotic signal that the cell perceives. These results highlight the importance of a systems approach to study the signaling networks.

## **Conclusions**

Our measurements of Hog1 phosphorylation dynamics in *C. albicans* suggest that, in this pathogen, Hog1 achieves perfect adaptation to a constant hyperosmotic shock through an integral control mechanism that is dependent on separation in time scales between signalling and downstream adaptation. We also predict that Hog1 signalling exhibits a short-term memory that can be revealed by tracking responses to an alternating signal. Furthermore, we predict that this short-term memory depends on the long-term memory. For example, Hog1 phosphorylation levels are predicted to adapt relatively quickly to high frequency signals in cells that have pre-adapted to a constant hyperosmotic stress.

## **Methods**

### **Strains and culture conditions**

*C. albicans* strain CA1655 (*ura3*::*λimm434*/*ura3*::*λimm434RPS1*/*rps1*::pACT1-FLAG-GFP) was used for the Hog1-phosphorylation assays. This strain is CAI-4 [39] containing pACT1-FLAG-GFP [40] integrated at the *RPS1* locus. *C. albicans* CA1655 expresses a FLAG-tagged version of GFP from the *ACT1* promoter and functions as an internal loading control for Western blots. *C. albicans* NGY152 [41], which is CAI4 transformed with CIp10 [42], was used for the glycerol assays. Strains were routinely maintained on YPD-T medium (2 % (w/v) glucose, 2 % (w/v) mycological peptone (Oxoid), 1 % (w/v) yeast extract (Oxoid), 100 mM Tris–HCl, pH 7.4) [43].

### **Cell growth and stress experiments**

Cells were grown at 30°C with shaking (200 rpm). To ensure consistency in the starting physiology of the cultures used in our experiments, cells were pre-cultured twice overnight in YPD-T, diluted to an OD_600_ of 0.2 in fresh medium and then grown to mid-log phase (OD_600_ 0.8) before any stress was applied [43]. A single hyperosmotic stress was applied by splitting one large starting culture and diluting the cells back to an OD_600_ of 0.2 in fresh YPD-T with NaCl added to achieve concentrations of 0, 0.3, 0.5, 0.75 or 1 M. Cells were harvested by centrifugation at selected time points and the pellets flash frozen in liquid nitrogen.

For the experiments involving the imposition of sequential stresses, cells were initially cultured and stressed as described above. At the end of the first stress period, cells were harvested, resuspended in fresh YPD-T with no NaCl and incubated at 30°C for 10 min. NaCl was then added to the cultures and samples were collected after the second stress period. Three biological replicates were prepared for each condition.

### **Protein extraction and western blotting**

To make protein extracts, flash-frozen cell pellets were resuspended in freshly prepared protein lysis buffer (50 mM Tris–HCl pH 7.5, 150 mM NaCl, 0.5 % NP40) containing inhibitors (2 mg/ml Leupeptin, 2 mg/ml Pepstatin, 1 mM PMSF, 2 mM Na_3_VO_4_ and 50 mM NaF) and sheared with acid-washed glass beads (425–600 μm, Sigma-Alrich) using a Fast-Prep FP120 machine (Thermo Scientific) (6 × 15 s bursts, Speed 6.5 with 1 min on ice between bursts). Protein extracts were clarified by centrifugation and protein concentration determined using a Bradford assay [44].

Proteins were separated by SDS-PAGE on NuPAGE®Novex Bis-Tris 4-12 % pre-cast polyacrylamide gels (Invitrogen, Paisley, UK). 15 μg protein was loaded in each lane. Proteins were transferred to PVDF membranes, rinsed in phosphate-buffered saline (PBS) and then blocked in PBS-T containing 10 % BSA (PBS, 0.1 % Tween20, 10 % (w/v) BSA) for 30 min at room temperature. Blots were incubated overnight at 4°C in PBS-T containing 5%BSA containing a 1:2000 dilution of anti-phospho-p38 MAPK (Thr180/Tyr182) antibody (Cell Signaling Technology) and a 1:20000 dilution of anti-FLAG antibody (Sigma-Aldrich, UK). Blots were washed in PBS-T and then incubated for 1 h at room temperature with Anti-rabbit IgG, HRP-linked antibody (Cell Signaling Technology). The blots were washed in PBS-T and the signal was detected using LumiGLO™ (Cell Signaling Technology) or SuperSignal®WestFemto (Thermo Scientific) according to manufacturer’s instructions. Phosphorylated Hog1 and GFP-FLAG levels were visualized and quantified using the FluorChem®FC2 (Alpha Innotech) or the FUSION SL™ (Peqlab) systems and the levels of phosphorylated Hog1 were expressed relative to the internal standard (GFP-FLAG).

### **Glycerol assay**

Extracellular and total glycerol concentrations were determined in stressed and unstressed cells at various time points. Cultures were grown and the stress applied as described above. The Free Glycerol Determination Kit (Sigma-Aldrich) was used to determine glycerol concentrations according to the manufacturer’s instructions. To measure extracellular glycerol concentrations, 1.5 ml samples of culture were collected and the cells harvested by centrifugation. Supernatants were transferred to a fresh tube and heated at 100°C for 10 min before their glycerol content was determined. Total glycerol concentrations were measured using 1.5 ml of culture after heating at 100°C for 10 min without prior removal of cells. Cell debris was spun down and the supernatant assayed for glycerol content. Intracellular glycerol concentrations were calculated by deducting the extracellular glycerol concentration from the total glycerol concentration.

## **Availability of supporting data**

Supporting data on the following topics are collated in an additional Supplemental data file with the manuscript:

Bioinformatic analysis of the conservation of proteins in HOG signaling network in *Candida albicans*

Mathematical modelling

· Steady-state response of HOG signaling network

· Frequency response of HOG signaling network

· Model assumptions

· The model

· Model parameterisation

· Frequency response of the system

## **Competing interests**

The authors declare that they have no competing interests.

## **Authors’ contributions**

Model development and analysis: TY, PI. Development of the repeated hyperosmotic shock experiment: APSM, MT, TY, MCR. Experimental data collection: MDJ, EC, MDL, AM. Data analysis: TY. Bioinformatic analysis of signaling component conservation: TT. Frequency response: TY, MCR, APSM, MT. Drafted the paper: TY. Also participated in paper writing: PI, MDJ, APSM, MCR, MDL, NARG, KH, AJPB. Supervision of modeling: CG, JS. Supervision of experiments: AJPB, KH, NARG. Conceived the research: JS, CG, MS, KH, AJPB. All authors approved the final manuscript.

## Supplementary Material

Additional file 1 A Systems Biology analysis of long and short-term memories of osmotic stress responses in fungi. Click here for file
