# Supplementary material for: A systems biology analysis of long and short-term memories of osmotic stress adaptation in fungi
Source: BMC Res Notes. 2012 May 25;5:258. doi: 10.1186/1756-0500-5-258 (PMC3434031; doi:10.1186/1756-0500-5-258)
Supplement: Additional file 1 — A Systems Biology analysis of long and short-term memories of osmotic stress responses in fungi. [file 1756-0500-5-258-S1.doc]

**Supplemental Data**

**A Systems Biology analysis of long and short-term memories of osmotic stress responses in fungi**

1. Bioinformatic analysis of the conservation of proteins in HOG signaling network in *Candida albicans*
2. Mathematical modelling
   1. Steady-state response of HOG signaling network
   2. Frequency response of HOG signaling network
   3. Model assumptions
   4. The model
   5. Model parameterisation
   6. Frequency response of the system

**1. Bioinformatic analysis of the conservation of proteins in HOG signaling network in *Candida albicans***


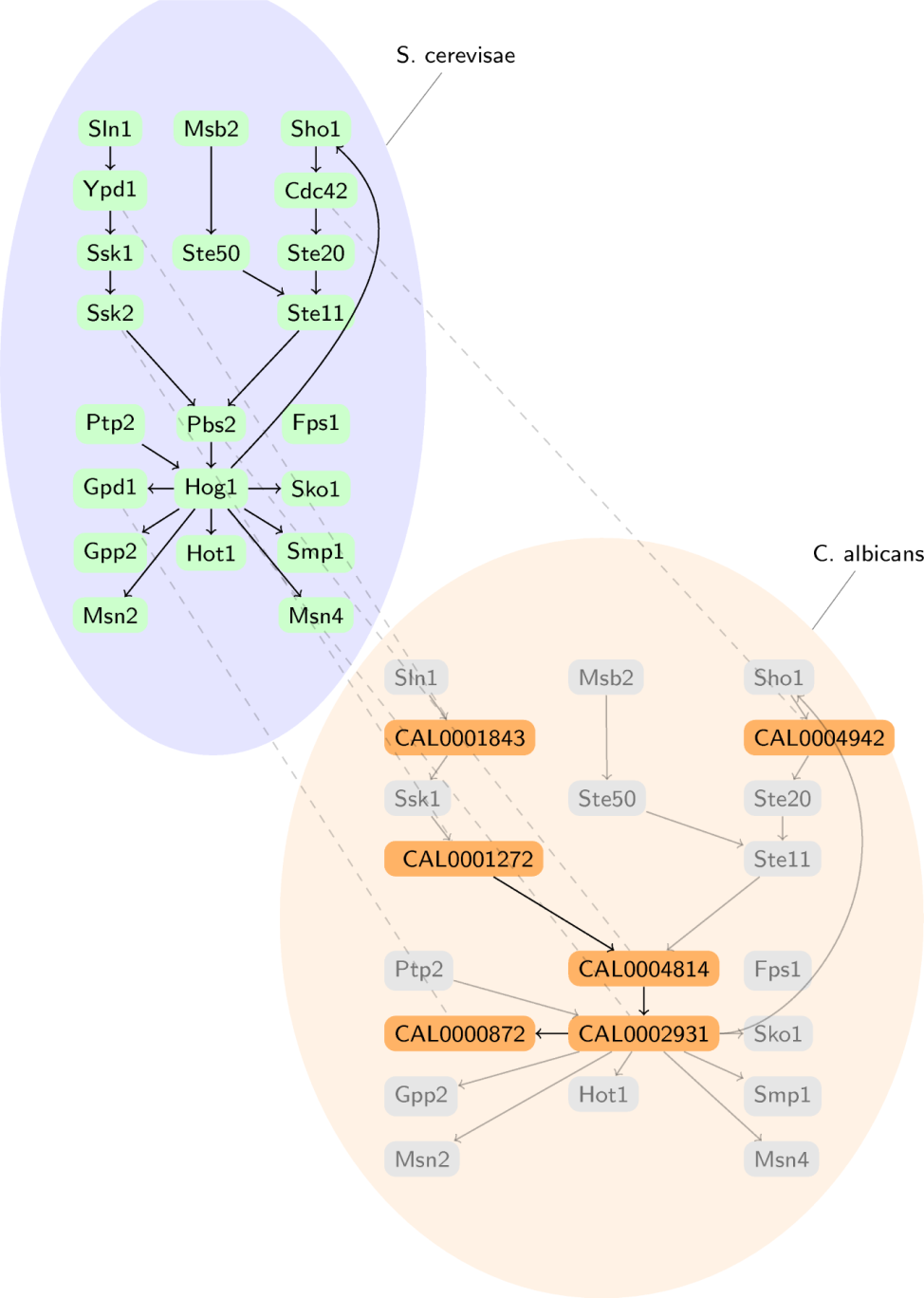


**Figure S1.** Conservation of proteins in the osmotic stress response pathway of *S. cerevisae* in *C. albicans*. Orthologous proteins were identified for each of the *S. cerevisiae* proteins in the pathway using a reciprocal BLAST methodology. Orthologues in *C. albicans* are highlighted in orange and labelled with their accession numbers, with dashed lines indicating their orthologues in *S. cerevisae*.

To assess the level of conservation of the elements of the currently known *S. cerevisiae* osmotic stress response pathway in *C. albicans* we performed a bioinformatics analysis to identify orthologous proteins between the species. Orthologues were detected using a reciprocal BLAST methodology, a standard approach in the literature, whereby a BLAST search is performed using the amino acid sequence of the *S. cerevisiae* protein in question into the *C. albicans* proteome, and where a match is found a reciprocal search is then performed back into the *S. cerevisiae* proteome. The pair of proteins identified are accepted as orthologues only if both are returned as the best hit in the BLAST search results of the other. As a cut-off for the BLAST hits we applied a sequence similarity cut-off of 30%, below which matches are not accepted, and require that the match covers at least 50% of the sequence of the smaller of the two proteins. This ensures that small regions of conserved sequence such as single domains are not incorrectly identified as being indicative of orthology between proteins. Our results show that whilst the core proteins involved in the response such as Pbs2, Hog1 and Gpd1 are conserved, the signalling pathways leading to them do not appear to have all their components intact. The majority of proteins thought to be involved in the regulatory response are also not conserved in *C. albicans,* with only Gpd1 present. It is worth mentioning that such an analysis depends on the definition of orthology used – it may be that there are proteins performing similar functions to those in *S. cerevisiae* present in *C. albicans*, but their sequences have diverged suitably far from their *S. cerevisiae* counterparts so as not to count as orthologues by our criteria.

**2. Mathematical modeling**

**2.1 Steady-state response of HOG signaling network**

Our goal is to construct a minimal network model that represents faithfully the osmotic responses in both fungi. The first step is to develop a concise model of HOG signal transduction network. There are several requirements of this part of the model. First, this signaling model should have at least a two-tier structure which allows activation of only a fraction of Hog1 when the signal saturates. In a two-tier enzymatic cascade, when upstream signaling component X is all phosphorylated at saturation signal, the fraction of phosphorylated Hog1 approximates a value that is determined by its own activation and inactivation activities, which is not necessarily 100%. This is important, since we found that in *C. albicans*, a typical hyperosmotic condition we discussed in this paper (i.e. 1M NaCl) leads Hog1 phosphorylation to plateau. However, a more severe hyperosmotic conditions that was assayed (2M NaCl, data not shown) triggered much higher Hog1 phosphorylation, presumably by other mechanisms not included in the current model. Hence, our model has a two-tier structure to describe signal relay.

Secondly, activation of Hog1 saturates at 0.2M NaCl in *S. cerevisiae*, as reported in a recent study that assayed Hog1 amplitude across different NaCl concentrations (Schaber *et al*, 2010). This should be reproduced by the signaling model.

Thirdly, the kinases of Pbs2, including Ste11, Ssk2 and Ssk22, are of much lower abundance than Pbs2, rendering a saturated activation likely (Gomez-Uribe *et al*, 2007). In addition, the phosphotases of Pbs2 (e.g. Ptc1-3) exist in equal or saturating amounts compared with Pbs2. Hence, Pbs2 is most likely operating under saturated activation and unsaturated inactivation *in vivo*. Such a regime allows Pbs2 to assume an ability to faithful transmit the signal in certain range without distortion *in vivo* (Gomez-Uribe *et al*, 2007). In addition, Hog1 induces the expression of its own phosphatase, Ptp2, Hog1 is likely to be operating under the same regime *in vivo*. As we demonstrate below, such regime (i.e. saturated activation and unsaturated inactivation) lead to undistorted signaling – the fraction of activated Pbs2 is proportional to the signal (elevation in extracellular osmotic pressure caused by NaCl) before the Pbs2 activation saturates, therefore in turn generates a linear response in Hog1. In our model, Michaelis-Menten kinetics instead of tQSSA is used. However, this does not change the property of this motif.


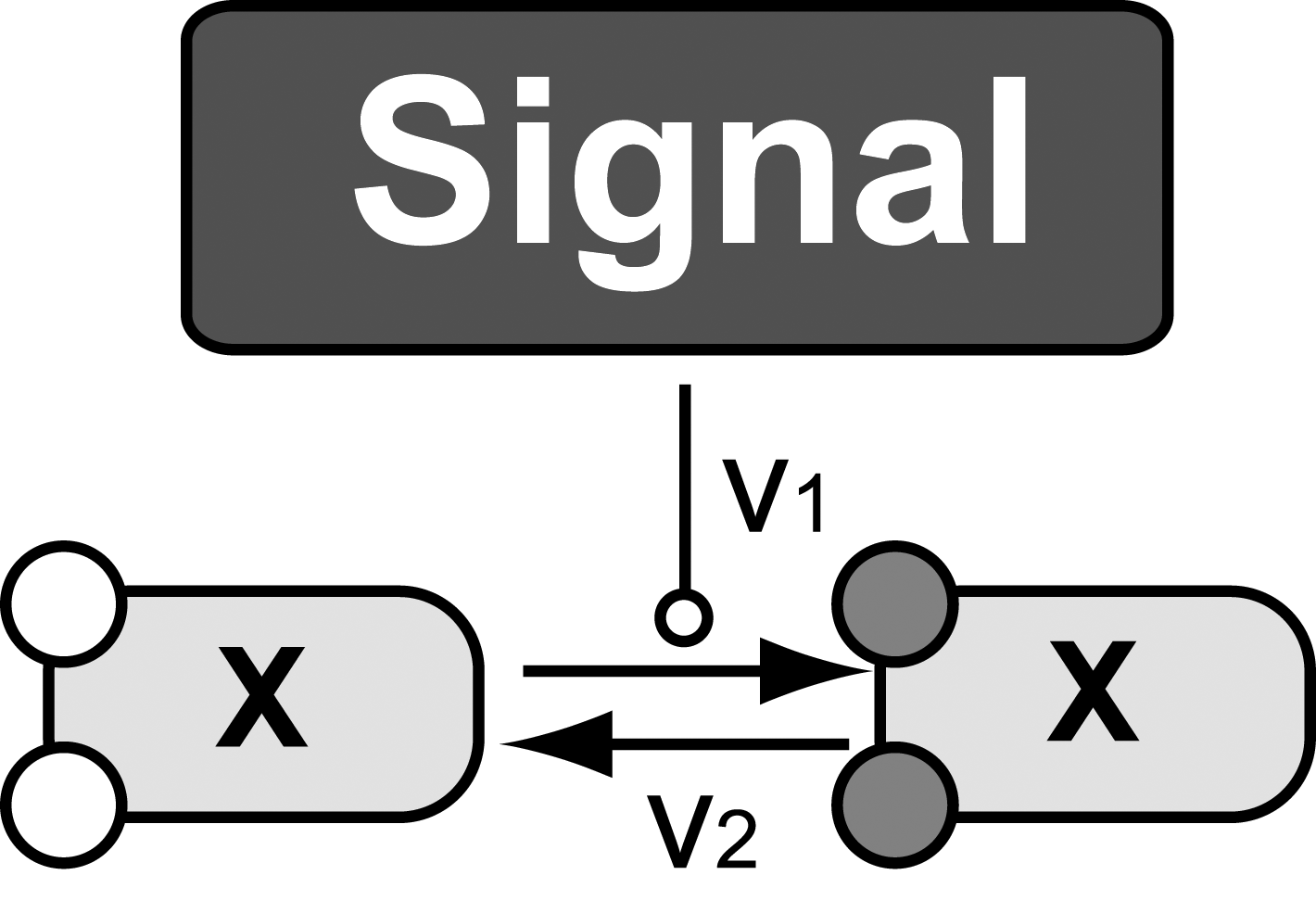


**Figure S2.** The diagram of a signal transduction motif. For the sake of discussion, we suppose the enzyme is activated via phosphorylation, while inactivated by dephosphorylation. The signal that activates the enzyme is abstract. It could be either a signal caused by hyperosmolarity as defined in the legend to Figure 2 in the main text, or an activated kinase of the enzyme. The enzyme is dephosphorylated by a phosphatase at a constant level.

Suppose this enzyme is governed under Michaelis-Menten kinetics,


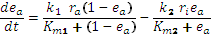
,

where
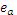
denotes the activated form of an enzyme with a total concentration of 1.
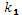
 and
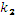
 are the catalytic rate constants of the kinase
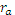
 and phosphatase
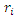
, respectively.
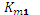
and
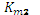
are Michaelis-Menten constants. The operation of an enzyme under saturated activation and unsaturated inactivation (i.e.
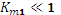
,
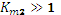
) can be approximated as


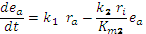


Setting the left-hand side of this equation to zero, the steady-state response is given by


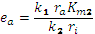
.

When the signal
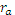
 saturates, almost all enzyme
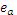
becomes activated (i.e.
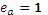
). Therefore, the saturation signal is
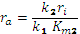
.

Figure S3 illustrates an example of the simplest signaling cascade. Suppose that the levels of phosphatases in reaction v_2_ and v_4_ are constant, and that both X and Hog1 are operating in the same regime of saturated activation and unsaturated inactivation (see legend of Figure S4 for kinetic parameter values). The upstream signaling component X reaches full activation at 0.2M NaCl, and consequently activates Hog1 at 50%.


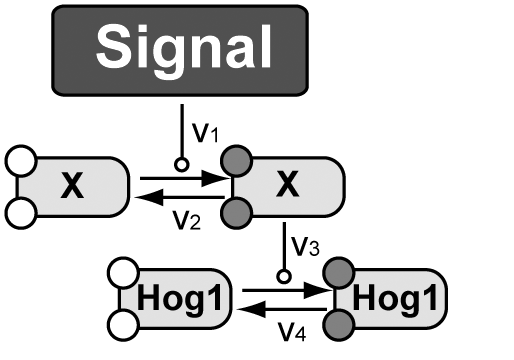


**Figure S3.** The diagram of a concatenated kinase cascade. For the sake of discussion, we suppose the enzymes are activated via phosphorylations, while inactivated by dephosphorylations.

Increasing k_1_ leads to an increase of the dose of NaCl at which X saturates. These results are important to construct the signaling network. However, the dynamic response to alternating signals is also useful in estimating appropriate kinetic parameters, as discussed in the next section.


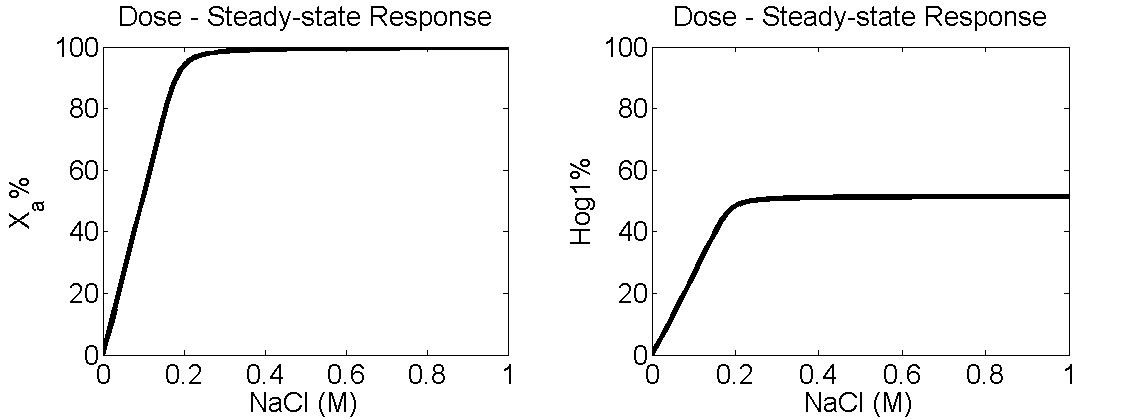


**Figure S4.** The steady-state response of a concatenated kinase cascade. The signal (identical to the extracellular NaCl concentration) activates an upstream signaling component X which in turn relays this signal to Hog1. Michaelis-menten kinetics is employed for both activation and inactivation of both components. For X, activation: k_1_=2.5, K_m1_=0.01; inactivation: k_2_=5, K_m2_=10. For Hog1, activation: k_3_=0.5, K_m3_=0.01; inactivation: k_4_=10, K_m4_=10. Both X_a_ and Hog1 saturate at 0.2M NaCl, but Hog1 plateaus at 50% activation.

**2.2 Frequency response of HOG signalling network**

Here, we begin our analysis by investigating how a signaling motif as shown in Figure S2 responds to oscillatory signal at a specific frequency. Suppose this signaling motif is regulated under Michaelis-Menten kinetics. We will deduce the frequency response of such a motif analytically. To our knowledge, it is the first time this is done.

$$\frac{dH}{dt}=\frac{k_{1}\cdot S\cdot\left(1-H\right)}{K_{1}+\left(1-H\right)}-\frac{k_{2}\cdot H}{K_{2}+H}$$

For the sake of simplicity, we consider a sinusoidal signal. Suppose the signal $S$fluctuates around a mean value $S_{0}$ by a small amplitude $A$,$S=S_{0}+A\cdot\cos\left(\omega t\right)$. Consequently, $H$fluctuates around a steady-state solution by a small deviation$H=H_{0}+\delta H$. The solution $H$can be expanded about $H_{0}$to 1^st^ order:

$$\begin{matrix} \frac{d\left(\delta H\right)}{dt}=\frac{k_{1}\cdot S\cdot\left(1-H_{0}-\delta H\right)}{K_{1}+\left(1-H_{0}-\delta H\right)}-\frac{k_{2}\cdot\left(H_{0}+\delta H\right)}{K_{2}+\left(H_{0}+\delta H\right)} \\ \approx\frac{k_{1}\cdot S\cdot\left(1-H_{0}\right)}{K_{1}+\left(1-H_{0}\right)}-\frac{k_{2}\cdot H_{0}}{K_{2}+H_{0}}-\left[\frac{k_{1}\cdot K_{1}\cdot S}{\left(K_{1}+1-H_{0}\right)^{2}}+\frac{k_{2}\cdot K_{2}}{\left(K_{2}+H_{0}\right)^{2}}\right]\delta H \end{matrix}$$

Now, substituting $S$:

$$\begin{matrix} \frac{d\left(\delta H\right)}{dt}\approx\frac{k_{1}\cdot\left[S_{0}+A\cos\left(\omega t\right)\right]\cdot\left(1-H_{0}\right)}{K_{1}+\left(1-H_{0}\right)}-\frac{k_{2}\cdot H_{0}}{K_{2}+H_{0}}-\left[\frac{k_{1}\cdot K_{1}\cdot\left(S_{0}+A\cos\left(\omega t\right)\right)}{\left(K_{1}+1-H_{0}\right)^{2}}+\frac{k_{2}\cdot K_{2}}{\left(K_{2}+H_{0}\right)^{2}}\right]\delta H \\ =\frac{k_{1}\cdot S_{0}\cdot\left(1-H_{0}\right)}{K_{1}+\left(1-H_{0}\right)}-\frac{k_{2}\cdot H_{0}}{K_{2}+H_{0}}+\frac{k_{1}\cdot A\cdot\cos\left(\omega t\right)\cdot\left(1-H_{0}\right)}{K_{1}+\left(1-H_{0}\right)}-\left[\frac{k_{1}\cdot K_{1}\cdot\left(S_{0}+A\cos\left(\omega t\right)\right)}{\left(K_{1}+1-H_{0}\right)^{2}}+\frac{k_{2}\cdot K_{2}}{\left(K_{2}+H_{0}\right)^{2}}\right]\delta H \end{matrix}$$

Because both $A$and$\delta H$are small, we ignore the higher order term$\frac{k_{1}\cdot K_{1}}{\left(K_{1}+1-H_{0}\right)^{2}}A\cos\left(\omega t\right)\delta H$, and noticing that $\frac{k_{1}\cdot S_{0}\cdot\left(1-H_{0}\right)}{K_{1}+\left(1-H_{0}\right)}-\frac{k_{2}\cdot H_{0}}{K_{2}+H_{0}}=0$ since $\frac{dH_{0}}{dt}=0$,

$\frac{d\left(\delta H\right)}{dt}\approx-\left[\frac{k_{1}\cdot K_{1}\cdot S_{0}}{\left(K_{1}+1-H_{0}\right)^{2}}+\frac{k_{2}\cdot K_{2}}{\left(K_{2}+H_{0}\right)^{2}}\right]\delta H+\frac{k_{1}\cdot\left(1-H_{0}\right)}{K_{1}+\left(1-H_{0}\right)}A\cos\left(\omega t\right)$.

Let $B=\frac{k_{1}\cdot K_{1}\cdot S_{0}}{\left(K_{1}+1-H_{0}\right)^{2}}+\frac{k_{2}\cdot K_{2}}{\left(K_{2}+H_{0}\right)^{2}}$,$C=\frac{k_{1}\cdot\left(1-H_{0}\right)}{K_{1}+\left(1-H_{0}\right)}A$, we have

$\frac{d\left(\delta H\right)}{dt}\approx-B\cdot\delta H+C\cos\left(\omega t\right)$.

Solving this equation, we have

$\delta H\left(t\right)=\left(\delta H_{0}-\frac{BC}{B^{2}+\omega^{2}}\right)e^{-Bt}+\frac{C}{\sqrt{B^{2}+\omega^{2}}}\sin\left(\omega t+\phi\right)$, where $\phi=\sin^{-1}\left(\frac{B}{\sqrt{B^{2}+\omega^{2}}}\right)$.

When time is large enough, the first term goes to zero, and$\delta H$oscillates by an amplitude of $\frac{C}{\sqrt{B^{2}+\omega^{2}}}$.

The amplitude of$\delta H$reaches its maximum$\frac{C}{B}$when $\omega=0$, and becomes zero when $\omega\to\infty$.

The amplitude of $\delta H$becomes half of its maximum at $\omega=\sqrt{3}B$.

We have evaluated the precision of this approximation by comparing the simulation results with the corresponding value predicted by the analytic approximation. We simulated the response of a two-tier kinase cascade as shown in Figure S3 (kinetic parameters in legend to Figure S4) to a sinusoidal signal that is centred at 0.1M NaCl with an amplitude of 0.05M NaCl. Frequency is sampled log uniformly from 0.001 to 10 rad/min (Figure S5). The approximation predicted higher amplitudes than the simulated results. In addition, the Hog1 amplitude is half of the corresponding amplitude of X. This is due to the choice of the parameters. As explained before, Hog1 plateaus at 50% when the signal saturates the activation of the system. Hence, a kinase cascade has a higher capacity to filter a fluctuating signal compared with a kinase motif. This property of the signaling pathway might be important for a cell that lives in a fluctuating environment.


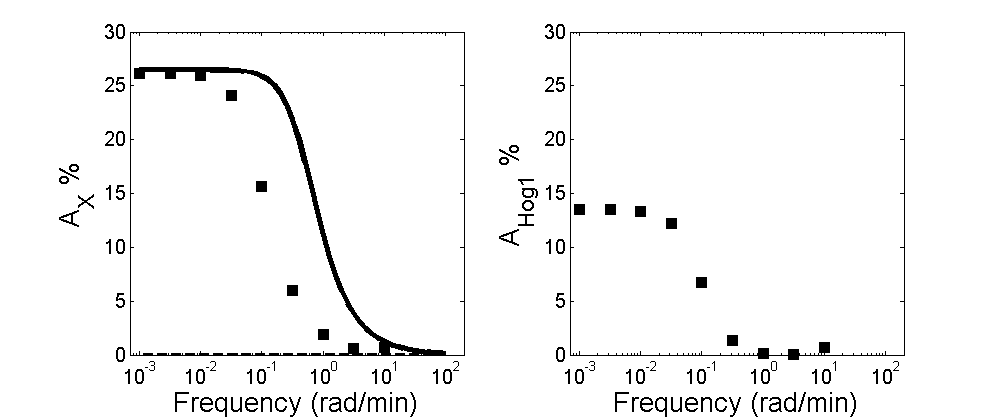


**Figure S5.** Frequency response of HOG signaling network. The two-tier cascade as shown in Figure S3 is excited by sinusoidal signal that oscillates around 0.1M NaCl with amplitude of 0.05M NaCl. The frequency varies from 0.001 to 10, sampled log uniformly. Kinetic parameter values are given in the legend to Figure S4. Analytical approximation: solid line; simulations: points.

An important character of the frequency response is the frequency at which amplitude is half of the maximum value (*i.e.* $\sqrt{3}B$). The analytical approximation (~12 rad/min) is about 4-times higher than the simulated value (~3 rad/min) (Figure S5, left panel). Hence, $\sqrt{3}B$ only draws an upper-bound of this value. This relation can be used to set an upper bound to the search space of kinetic parameters during model parameterisation.

**2.3 Model assumptions**

As mentioned in the text, the process that transmits hyperosmotic stress signal and results in double phosphorylation of Hog1 in *C. albicans* is not completely understood. For example, it is not known whether hyperosmotic stress causes inhibition of HOG signalling inactivation or induces activation of HOG signalling. The lack of knowledge necessitates a higher level abstraction when this process is modelled. Here, we propose that the osmotic stress signal induces HOG signalling.

Hog1 → Hog1-PP (v_1_, catalysed by signal) 2.3.1

A natural question that follows immediately is the nature of the signal that is sensed by the cell and causes osmotic shock responses. There are several possibilities. Considering the functions of the postulated osmotic sensors, the stress signal might be the change in turgor pressure (Sln1’s postulated role), the absolute value of extracellular osmotic pressure (Hkr1 and Msb2’s postulated role), or both (depending on the presence and functionality of these proteins in a species). The calculation of turgor pressure involves the measurement of volume and the mechanical properties of the cell wall, which are both hard to quantify and are left outside the scope of the model. To avoid these difficulties, we consider the process of adaptation instead. We assume that the entire system adapts when turgor pressure is fully restored. Under the new steady state, the signal should reduce to zero as Hog1-pp comes back to the basal level. Conforming to this requirement, we propose

$S\left(t\right)=\left(\Pi_{i}^{0}-\Pi_{e}^{0}\right)-\left[\Pi_{i}\left(t\right)-\Pi_{e}\left(t\right)\right]$ 2.3.2

The item in the first brackets equals to the initial turgor pressure prior to stress. The item in the second brackets is the difference between intracellular and extracellular osmotic pressure for a hypothesised cell with invariant cell volume (in a real cell that changes volume, this difference should reduce to zero immediately after the onset of hyper-osmotic shock due to the fast efflux of water). The signal *S*, being the difference between the two items, is the pressure needed to fully restore the turgor pressure. Although the coarse grain model does not consider volume changes and the definition of the signal does not deal with turgor pressure, the signal implicitly expresses the fact that turgor pressure should be restored once the system adapts, which is supported by a recent publication (Muzzey *et al*, 2009).

The next question is what reaction kinetics should be employed to relate *S* with Hog1 phosphorylation rate. Simulation of a subset of relevant equations from Klipp-Hohmann model that describe signal processing reveals the relationship between turgor pressure (*i.e.* the signal in that model) and the nuclear Hog1-PP (*i.e.* the output of the entire signal processing pathway) in the workings of that model (Figure 1.4.1). This clearly demonstrated a sigmoidal relationship between the two, and suggests a saturating kinetics should be employed in the coarse grain model. The simplest form such kinetics is Michaelis-Menten kinetics that requires two kinetic parameters. As mentioned previously, we use a two-tier kinase cascade employing Michaelis-Menten kinetics to describe signal transduction. The total concentrations of X and Hog1 are assumed to be 1.

**
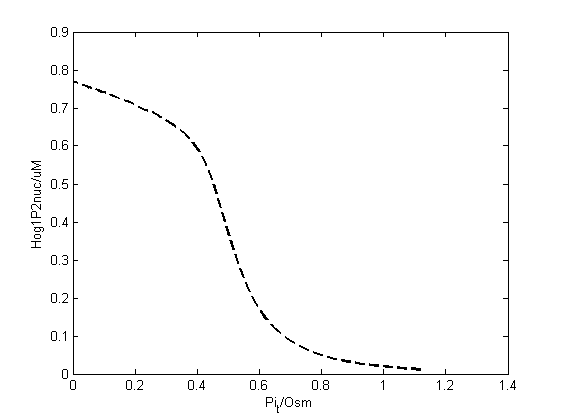
**

**Figure S6.** Simulation results of a subset of relevant equations from Klipp-Hohmann model reveals the relationship between turgor pressure and nuclear Hog1-pp captured in that model. The simulations were carried out for a subset of equations including biophysical change in the system, upstream *SLN1* branch sensing of the change in turgor pressure, MAPK signalling cascade, phosphorylation-dependent of Hog1 transport into the nucleus, Hog1-dependent Ptp2 phosphotase expression, and Hog1 dephosphyrlation by Ptp2. To generate the simulations, Π_e_ was surveyed in the range spanning from 0.1 to 10 Osm (100 conditions are uniformly sampled). The steady-state nuclear Hog1-pp level is plotted against the corresponding turgor pressure.

In *S. cerevisiae*, phosphorylated Hog1 is dephosphorylated in a Ptp2-dependent fashion, and Hog1 activity induces the expression of *PTP2* gene. For simplicity, Ptp2 is not included explicitly in our model. Hence, a constant Ptp2 activity is implicitly assumed.

**Glycerol production mediated by Hog1**

Active Hog1 induces the expression of *GPD1* and *GPD2* genes that encode enzymes catalysing the production of glycerol in *S. cerevisiae*. However, this entire production process relies on a complex pathway as described in the Klipp-Hohmann model, and the relationship between active Hog1 (and Gpd1, Gpd2 protein levels) and glycerol production rate is obscure. To explore the possibility of approximating how active Hog1 affects glycerol production in Klipp-Hohmann model using less equations, we first investigated the relationship between Gpd1 (Gpd2) levels with glycerol production rate (Klipp *et al*, 2005). In that model, the concentration of Gpd1 and Gpd2 enzymes was represented by an abstract variable known as Protein 1. The simulation results demonstrate glycerol biosynthesis rate is roughly proportional to Hog1-dependent enzyme activities in a wide range (Figure S7). Hence, it suggests that the expression levels of Hog1-dependent glycerol biosynthetic enzymes may be representative of glycerol production capacity in *S. cerevisiae*.


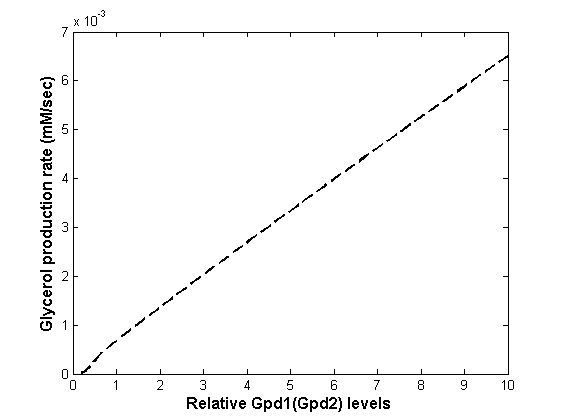
**Figure S7.** The relationship between Gpd1 (Gpd2) levels with glycerol production rate in Klipp-Hohmann model. The metabolism module of that model was simulated, assuming a constant Gpd1 (Gpd2) protein concentration. The range between 10% to 10-fold upregulation of the relative concentration compared with the initial concentration was investigated (100 conditions are uniformly sampled). Volume change was ignored. The steady-state glycerol production rate is plotted against the relative Gpd1(Gpd2) levels.

We experimentally monitored Gpd1 levels in *C. albicans* upon hyperosmotic shock. We found that this enzyme has a high background expression in the absence of hyperosmotic shock, and it is not significantly upregulated under underosmotic stress. In contrast, *GPD2* mRNA was found to increase significantly in response to hyperosmolarity. This suggests that modulation of glycerol production in *C. albicans* may happen at different steps. Because we do not directly measure glycerol production rate *in vivo*, we used a variable called glycerol production rate in our model.

We also investigated the basal glycerol production that is independent from Hog1. The *hog1∆ C. albicans* mutant does not accumulate intracellular glycerol under hyperosmotic conditions (data not shown). This rules out the possibility of a significant HOG-independent glycerol production. Hence, we assumed that the glycerol production rate is entirely Hog1-dependent in *C. albicans*.

**2.4 The Model**

Variables

1. *X*: active form of the upstream signaling component (*X* + *X_i_* =1)
2. *H*: active Hog1 (dimensionless, *H* + *H_i_* =1)
3. *P_G_*: glycerol production rate (mOsM/(g∙min))
4. *G_i_*: absolute amount of intracellular glycerol (mOsM/g)
5. *G_e_*: absolute amount of extracellular glycerol (mOsM/g)

Intermediate variables

1. *X_i_*: inactive X (dimensionless, *X* + *X_i_* =1)
2. *H_i_*: inactive Hog1 (dimensionless, *H* + *H_i_* =1)

Biochemical equations

1. *X_i_* → *X* regulated by signal *S*
2. *X* → *X_i_* catalysed by a constant phosphatase
3. *H_i_* → *H* regulated by X
4. *H_i_* → *H* catalysed by a constant phosphatase
5. → *P_G_* increase in glycerol production rate induced by active Hog1
6. *G_i_* → *G_e_* passive diffusion of glycerol driven by concentration difference
7. *P_G_* → decay in glycerol production rate under hypoosmotic condition

State-dependent parameters

1. $\Pi_{i}=G_{i}+n$ (mOsM/g)

Intracellular osmotic pressure Π*_i_* is exherted by *G_i_* (intracellular glycerol) and *n* (other substance that contribute to osmolarity). Here, we assume that intracellular volume for osmotic substance is 1. Hence, the osmolarity exerted by intracellular glycerol is equivalent to *G_i_*.

1. $S\left(t\right)=\left(\Pi_{i}^{0}-\Pi_{e}^{0}\right)-\Pi_{i}\left(t\right)-\Pi_{e}\left(t\right)$ (mOsM/g)

This signal dictates phosphorylation rate of X.

1. $c_{G}=\max\left(-k_{AGP}\cdot S+b_{AGP},0\right)$ (dimensionless)

The degree of opening of the glycerol channels. 0 refers to full closure. $b_{AGP}$ defines the resting state.

Reaction rate equations

$$v_{1}=\frac{k_{1}\cdot S\cdot\left(1-X\right)}{K_{m1}+\left(1-X\right)}$$

$$v_{2}=\frac{k_{2}\cdot X}{K_{m2}+X}$$

$$v_{3}=\frac{k_{3}\cdot X\cdot\left(1-H\right)}{K_{m3}+\left(1-H\right)}$$

$$v_{4}=\frac{k_{4}\cdot H}{K_{m4}+H}$$

$$v_{5}=k_{5}\cdot H$$

$$v_{6}=c_{G}\cdot\left(G_{i}-\frac{G_{e}}{V_{e}}\right)$$

$$v_{7}=k_{7}\cdot P_{G}$$

Initial conditions

|  |  | *S. cerevisiae* | *C. albicans* |
| --- | --- | --- | --- |
| 1 | $X$(dimensionless) | 0 * | 0 * |
| 2 | $H$(dimensionless) | 0 * | 0 * |
| 3 | $P_{G}$(mOsM/(g∙min)) | 0 * | 0 * |
| 4 | $G_{i}$(mOsm/g) | 0.002 (Gennemark) | 0 * |
| 5 | $G_{e}=G_{i}\times V_{e}$(mOsm/g) | 2 | 4 |
| 6 | $n=\Pi_{i}-G_{i}$(mOsm/g) | 0.638 | 1 |
| 7 | $V_{e}$(dimensionless) | 1000 * | 1000 * |
| 8 | $\Pi_{i}$(mOsm/g) | 0.64 (Gennemark) | 1 * |
| 9 | $\Pi_{e}$(mOsm/g) | 0.24 (Gennemark) | - 1. (Emily Cook) |

* hypothesised value

Parameters (significance of all values is 1)

|  |  | *S. cerevisiae* | *C. albicans* |
| --- | --- | --- | --- |
| 1 | $k_{1}$( g/mOsM) | 2.5 | 0.5 |
| 2 | $K_{m1}$(dimensionless) | 0.01 | 0.01 |
| 3 | $k_{2}$(dimensionless) | 5 | 5 |
| 4 | $K_{m2}$(dimensionless) | 10 | 10 |
| 5 | $k_{3}$(dimensionless) | 0.5 | 0.5 |
| 6 | $K_{m3}$(dimensionless) | 0.01 | 0.01 |
| 7 | $k_{4}$(dimensionless) | 10 | 10 |
| 8 | $K_{m4}$(dimensionless) | 10 | 10 |
| 9 | $k_{5}$(mOsM/(g∙min)) | 0.005 | 0.007 |
| 10 | $k_{AGP}$ | 0.02 | 0.8 |
| 11 | $b_{AGP}$ | 0.05 | 0.08 |
| 12 | $k_{7}$(/min) | 0.03 * | 0.03 * |
| 13 | $P_{b}$(mOsM/(g∙min))** | 0.02 | 0 |

* It was assumed that glycerol production rate does not decay in response to hyperosmotic conditions. In other words, $k_{7}$is 0 under hyperosmotic conditions. In response to hypo-osmotic condition, glycerol production rate decreases in a first-order reaction with a half-life of 20 minutes. The value of ln2/20 (~0.03) is used in simulations (it is used to generate Figure 4 *etc*).

** Basal glycerol production that is Hog1-independent.

ODE’s

$$\frac{dX}{dt}=\frac{k_{1}\cdot S\cdot\left(1-X\right)}{K_{m1}+\left(1-X\right)}-\frac{k_{2}\cdot X}{K_{m2}+H}$$

$$\frac{dH}{dt}=\frac{k_{3}\cdot X\cdot\left(1-H\right)}{K_{m3}+\left(1-H\right)}-\frac{k_{4}\cdot H}{K_{m4}+H}$$

$$\frac{dP_{G}}{dt}=k_{5}\cdot H-k_{7}\cdot P_{G}$$

$$\frac{dG_{i}}{dt}=P_{G}+P_{b}-c_{G}\cdot\left(G_{i}-\frac{G_{e}}{V_{e}}\right)$$

$$\frac{dG_{e}}{dt}=c_{G}\cdot\left(G_{i}-\frac{G_{e}}{V_{e}}\right)$$

The decay in $P_{G}$ is assumed to happen in a much slower time-scale than that of signal transduction under hyperosmotic conditions. Hence, we can ignore the decay term in (3) under this condition, and this equation reduces to $\frac{dP_{G}}{dt}=k_{5}\cdot H$. Here, $P_{G}$ integrates over $H$. In addition, $P_{G}$ positively controls$G_{i}$, which attenuates the signal *S*. This constitutes a negative feedback loop that contains an integral controller, a case similar to Box 1C in the text. In this way, the integral control in osmoregulation arises from time-scale separation.

**2.5 Model parameterisation**

Most of the kinetic parameters are from the literature, or are estimated based on the hypotheses as described and examined in the previous subsections. Only 3 parameters $k_{5}$, $k_{AGP}$, and $b_{AGP}$ are manually chosen to reproduce single shock experimental data, including time course results of Hog1 phosphorylation, intracellular and total glycerol concentrations in relative values. These data are tabulated in the supplemental excel files. Each set of data and simulation results is normalised against the highest value in this set (either data or simulation) before comparison.

**2.6 Frequency response of the system**

As discussed in the main text, the system’s response to a high frequency sinusoidal signal depends on both HOG signal transduction network and the regulation of aquaglyceroporins. During an “off” phase, if aquaglyceroporin is able to respond instantaneously to the hypo-osmotic condition by releasing intracellular glycerol, the cell would lose its memory of the previous hyperosmotic shock and treat the next hyperosmotic shock the same as it does before. Thus, phosphorylation of Hog1 eventually oscillates around a constant level (Figure 6A). However, if glycerol release is slower, the cell would maintain glycerol from previous hyperosmotic shocks, and the phosphorylation of Hog1 would oscillate around a lower level. This prediction is reproduced by our simulation of a *C. albicans* cell with $k_{AGP}$ set to 0.5. It is worth noting that the behaviour of Hog1 under a constant signal is also quantitatively affected by the value of $k_{AGP}$ - the inflexion point in Figure S8A is more consipicuous compared with Figure 6A.


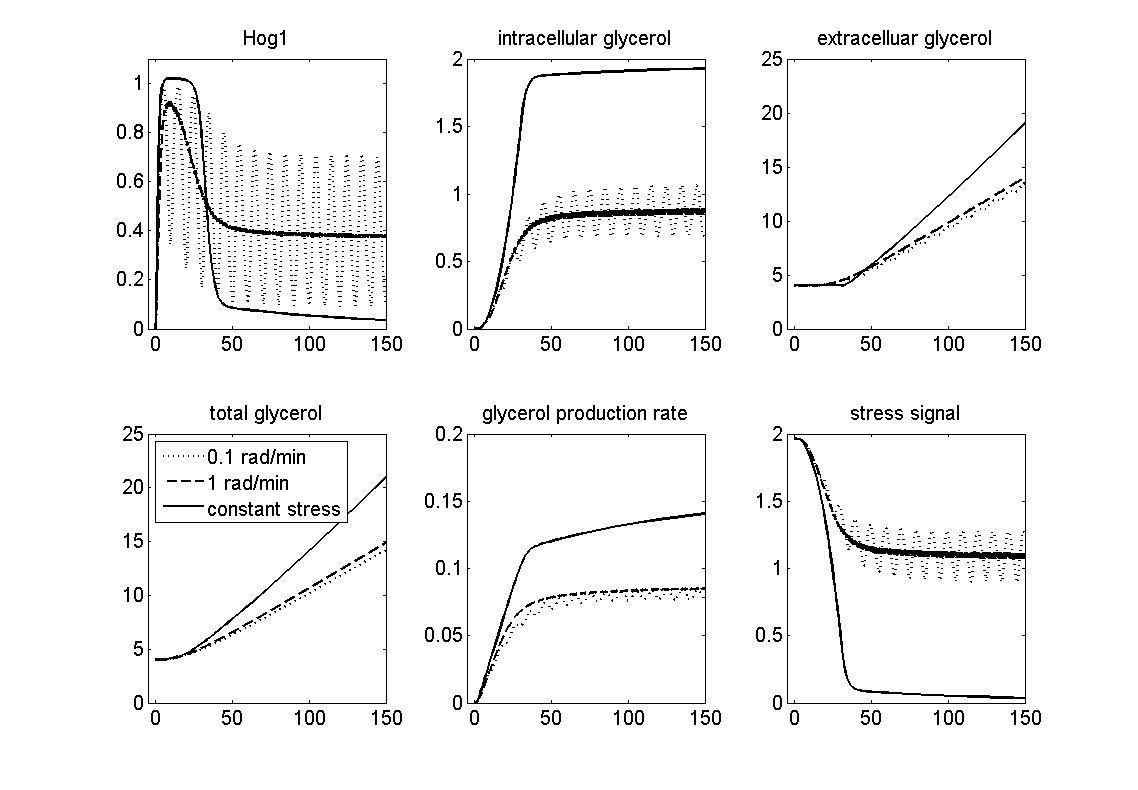


**Figure S8.** Frequency response of the system. Responses of a *C. albicans* cell to a sinusoidal signal with peak-to-peak amplitude of 1M NaCl (centred at 0.5M NaCl) at different frequencies are simulated (dotted line: 0.1 rad/min; dashed line: 1 rad/min; real line: constant stress). Different from Figure 6 in the main text, $k_{AGP}=0.5$.

**Reference**

Gomez-Uribe C, Verghese GC, Mirny LA (2007) Operating regimes of signaling cycles: statics, dynamics, and noise filtering. *PLoS Comp Biol* 3(12): 2487-2497.

Mettetal JT, Muzzey D, Gómez-Uribe C, van Oudenaarden A. (2008) The frequency dependence of osmo-adaptation in *Saccharomyces cerevisiae*. *Science*. 319(5862): 482-484.

Muzzey D, Gómez-Uribe C, Mettetal JT, van Oudenaarden A. (2009) A systems-level analysis of perfect adaptation in yeast osmoregulation. *Cell*. 138: 160-171.

Schaber J et al (2010) Biophysical properties of Saccharomyces cerevisiae and their relationship with HOG pathway activation. *Eur Biophys J*
